# 10 Years of Human-NAO Interaction Research: A Scoping Review

**DOI:** 10.3389/frobt.2021.744526

**Published:** 2021-11-19

**Authors:** Aida Amirova, Nazerke Rakhymbayeva, Elmira Yadollahi, Anara Sandygulova, Wafa Johal

**Affiliations:** ^1^ Graduate School of Education, Nazarbayev University, Nur-Sultan, Kazakhstan; ^2^ Department of Robotics and Mechatronics, School of Engineering and Digital Sciences, Nazarbayev University, Nur-Sultan, Kazakhstan; ^3^ École Polytechnique Fédérale de Lausanne (EPFL), Lausanne, Switzerland; ^4^ University of Melbourne, Melbourne, VIC, Australia; ^5^ UNSW, Sydney, NSW, Australia

**Keywords:** social robot, human-robot interaction, nao, survey, review, humanoid robot, qualitative, quantitative

## Abstract

The evolving field of human-robot interaction (HRI) necessitates that we better understand how social robots operate and interact with humans. This scoping review provides an overview of about 300 research works focusing on the use of the NAO robot from 2010 to 2020. This study presents one of the most extensive and inclusive pieces of evidence on the deployment of the humanoid NAO robot and its global reach. Unlike most reviews, we provide both qualitative and quantitative results regarding how NAO is being used and what has been achieved so far. We analyzed a wide range of theoretical, empirical, and technical contributions that provide multidimensional insights, such as general trends in terms of application, the robot capabilities, its input and output modalities of communication, and the human-robot interaction experiments that featured NAO (e.g. number and roles of participants, design, and the length of interaction). Lastly, we derive from the review some research gaps in current state-of-the-art and provide suggestions for the design of the next generation of social robots.

## 1 Introduction

For some decades, social robots have been used for research purposes in an attempt to assist humans and bring social benefits to their life. These social robots have been envisioned to interact with humans in various application domains such as education, healthcare, industry, entertainment, and public service. However, in order to claim that social robots reached their full potential as social assistive agents, they have to be able to create sustainable and intelligent interactions in the real world while acting in an acceptable and credible way. Therefore, the field of human-robot interaction has fueled research into the design, development and evaluation of social robots. There is a significant number of social robots in research, such as Kaspar for autism therapy (Wood et al., 2019), iCub for cognitive development (Natale et al., 2016), and Robovie for public spaces (Das et al., 2015), and the NAO robot. NAO has been among the most widely used social robots in human-robot interaction research due to its affordability and broad functionality. Developed by the French company, Aldebaran Robotics, in 2008 and acquired by the Japanese company, Softbank Robotics, in 2015, NAO is an autonomous and programmable humanoid robot that has been successfully applied to research and development applications for children, adults, and the elderly people. More than 13,000 NAO robots are used in more than 70 countries around the world. Consequently, a number of recent large-scale interdisciplinary projects, such as ALIZ-E^1^, DREAM^2^, CoWriter^3^, SQUIRREL^4^, L2Tor^5^ have explored child-centered research with the mission to enable NAO to take a role of a tutor, a therapist, or a peer learner.

There have been several reviews about social robots used for specific application domains, such as robot-assisted education (Mubin et al., 2013; Belpaeme et al., 2018; Johal, 2020) and autism therapy (Saleh et al., 2020). There is evidence that NAO was among the heavily used social robots for these applications (Belpaeme et al., 2018; Saleh et al., 2020; Henschel et al., 2021). Among the most recent literature surveys, Robaczewski et al. (2020) reviewed the use of NAO as a socially assistive robot (SAR). The authors studied a total of 51 user-study publications and discussed their major findings around six themes: social engagement, affectivity, intervention, assisted teaching, mild cognitive impairment/dementia, and autism/intellectual disability. While providing a good overview of some of the social assistive robotics studies that were conducted with the NAO, this previous survey does not consider technical contributions, thus is limited in identifying research and development trends in its deployment across application domains. Therefore, it is still unclear how and why this social robot has been used in research over the last 10 years and how this standardized platform contributed more widely to the field of human-robot interaction.

For these reasons, a scoping review was a necessary step to systematically map the research done with the NAO robot in HRI and identify research trends and potential gaps of investigations that could lead to the development of a new standard platform for social robotics research. It seems a worthwhile effort to reflect on the dynamics of the socially acceptable robot - a humanoid NAO robot - that has a particular appeal for improving the social, behavioral, physical, and cognitive well-being of humans of various age groups. The present paper aims to provide a holistic understanding of the NAO robot for research by analyzing the unrestricted type of contributions, both theoretical and experimental. We also report on technical contributions that helped the field of HRI to grow over the years. While following a strict and reproducible protocol, our review probably does not cover the complete literature work in HRI research with the NAO robot. However, we consider that our screening protocol allowed to capture a good amount of the body of research using NAO and to present useful insights, findings, and trends in the use of the robot in the past decade. Unlike previous reviews, our research approach allows us to present general and specific findings that were gleaned from quantitative and qualitative analysis. We find our review vital in understanding how the social robots like NAO serve educational, professional, and social roles when interacting with humans and what are the crucial insights about its use and prospects. This research potentially benefits a wider community of stakeholders such as novice and expert HRI researchers, robotics labs or startups and those professionals working at the intersection of interdisciplinary fields like education and healthcare.

Our meta-analysis seeks to provide broad insights into the use of NAO in HRI by annotating a wide range of categories of applications (including but not limited to social assistive robotics), geographical distribution, type of contribution, application fields, experimental methodology, duration, and the number of sessions, human-robot ratio, participant demographics, human-robot roles, robot autonomy, input/output data, and equipment used. We propose respectively: a quantitative analysis allowing to observe objective metrics on trends and qualitative analysis of the relevant research topics to HRI covered by papers used in this review.

## 2 Technical Overview of NAO Over the Years

NAO is 58 cm in height and weighs 5.6 kg. The robot is programmed by a specialised NAOqi framework, has an easy to use graphical programming tool Choregraphe (for complex applications and control of motions), and Monitor (for robot feedback and verification of joints or sensors), all of which allow to easily program and introduce the NAO behaviours (Bertacchini et al., 2017). It can be connected *via* wired or wireless (Wi-fi) network, thus allowing autonomous operation and remote control, which is important, especially when the robot is operating in a real-world setting. It has 25° of freedom, of which 12 for legs, five for the arms, two for the head, which enables it to move and perform actions. Furthermore, it has four directional microphones and speakers and two cameras that are necessary for basic modules such as built-in text-to-speech and speech recognition for 20 languages, object recognition, face detection, recognition, and tracking, all of which provide the possibility to act more naturally and human-like. Table 1 presents an overview of NAO’s hardware and software improvements over the years. For example, NAO’s V3 in 2008 supported only nine languages, while the current V6 version provides support for 20 languages. Additionally, NAO’s cameras, microphones, and storage were improved in three instances: from V3 to V4 or V5 to V6.

**TABLE 1:** NAO’s evolution in technical characteristics over the years.

NAO version	V3+ (2008)	V3.2 (2009)	V3.3 (2010)	V4 (2011)	V5 (2014)	V6 (2018)
Storage	2 GB Flash memory			2 GB+8 GB Micro SDHC		32 GB SSD
2 × Cameras	640 × 480, 30 fps			1280 × 960, 30 fps		640 × 480, 30 fps or 2560 × 1920, 1 fps
	58 Diagonal Field Of View			72.6 Diagonal FOV		
	(47.8 Horizontal FOV, 36.8 Vertical FOV)			(60.9 Horizontal FOV, 47.6 Vertical FOV)		67.4 Diagonal FOV (56.3 Horizontal FOV, 43.7 Vertical FOV)
4 × Microphones	Sensitivity: −40 mV/Pa ± 3 dB				20 mV/Pa ± 3dB	Omnidirectional
						250 mV/Pa ± 3dB
						100 Hz to 10 kHz
	Frequency range: 20 Hz–20 kHz				150 kHz to 12 kHz	
Languages	9 (English, French, Spanish, German, Italian, Japanese, Korean, Chinese, Portuguese)			19 languages (+ Arabic, Czech, Danish, Dutch, Brazilian, Greek, Polish, Finnish, Swedish, Russian, Turkish		20 languages (+ Norwegian)

The first NAO driver for Robot Operating System (ROS) was released by Brown University’s RLAB in November of 2009 (ROS, 2010) which supported head control, text-to-speech, basic navigation, and access to the cameras. Later, the University of Freiburg’s Humanoid Robot Lab improved NAO’s driver with new capabilities, such as torso odometry and joystick-based teleoperation. Already in December that year, the Humanoid Robot Lab released a complete ROS stack for the NAO that additionally contained IMU state, a URDF robot model, visualization of the robot state in rviz, and more (ROS, 2010).

Additionally, NAO users around the world had an opportunity to download an existing behavior or upload their own robot behavior to the Application Store. In 2014, ASK NAO^6^ was released to support ready robot behaviors for conventional and special education. Similarly, but with a more general purpose, Zora Solution Software^7^ was also offered to the market with more than 50 different robot activities to be used *via* a tablet by a non-technical user (such as a health professional).

## 3 Methodology

**FIGURE 1:**
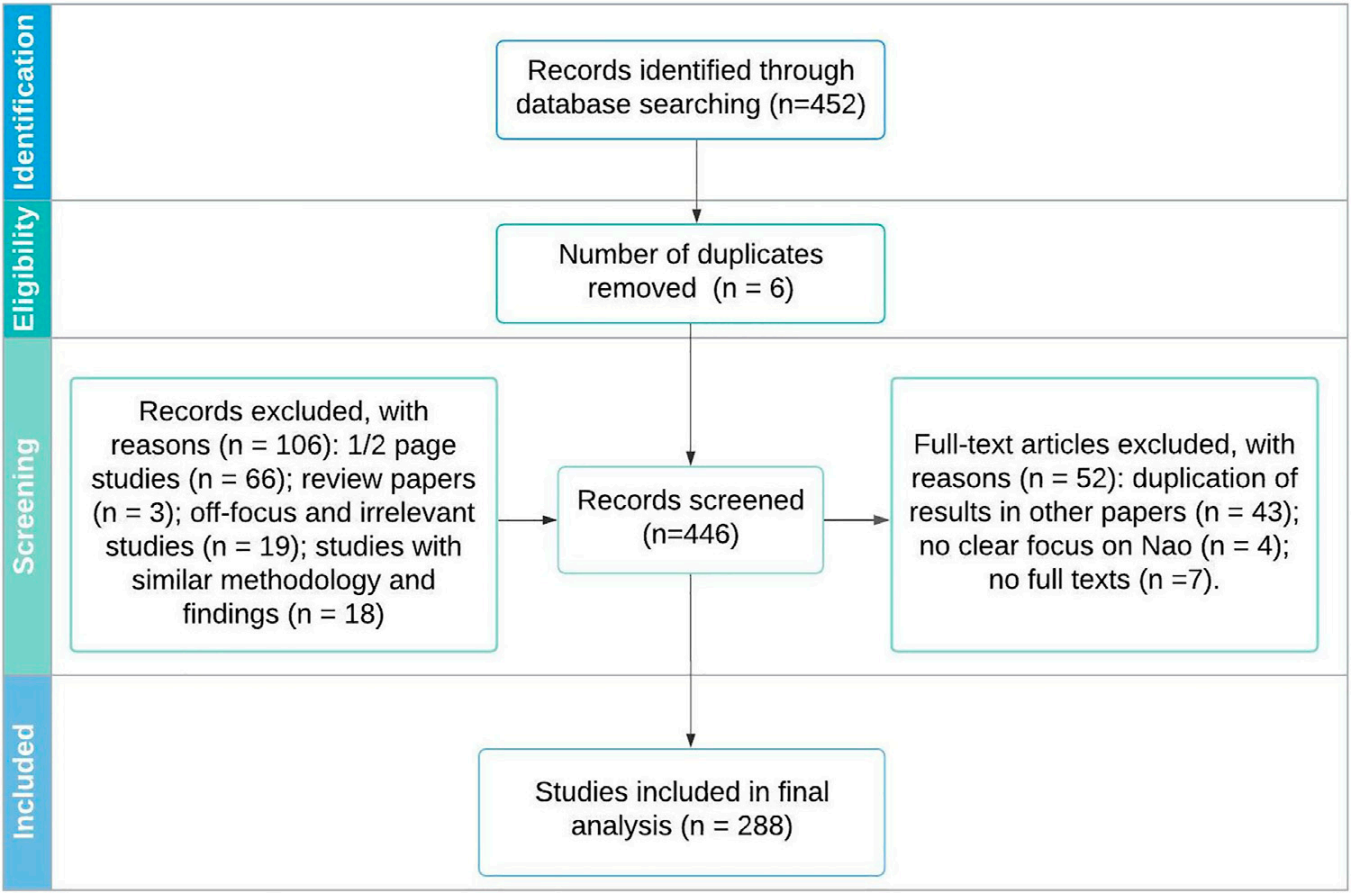
The screening process (adapted from PRISMA template 2009).

Our methodology followed similar works previously published in HRI and presenting a review of articles in the domain (Belpaeme et al., 2018; Johal, 2020; Obaid et al., 2020). We adopted a scoping review framework to extract relevant information from the literature to address our research questions. This approach is helpful to provide an overview of diverse research evidence in broad types of literature (Sucharew and Macaluso, 2019). We describe below the procedure carried out to collate the set of the relevant article and analyze their content in Figure 1 which follow the PRISMA flowchart.

### 3.1 Identification

To identify potentially relevant documents, the Scopus^8^ bibliographic database was searched for papers published from 2010 to October 2020. The term search was performed in October 2020. The Scopus database includes IEEE, Springer, and ACM DL and allows it to cover a wide range of publication venues. Because our goal is to broadly look at the research works done in HRI with NAO, we kept the search term open. We limited our search string to English-written publications as we searched for the terms “NAO” AND “human-robot interaction” in title, abstract, or keywords.

Overall, an initial 452 records were retrieved and underwent the screening process. They were stored on Zotero and then were exported into BibTeX and CSV. The following steps of the analysis of the collected documents were done by entering information on an online Google spreadsheet.

### 3.2 Screening Process

After identifying the records, we first consulted abstracts to ensure that they used NAO in the study. We excluded 106 studies provided only a quick overview (e.g., workshop, demonstration) in one or two pages in length. We removed the review and off-topic papers that lack any NAO intervention, both theoretically and practically.

In the second round, we consulted full texts to ensure that the chosen records do not replicate results. Since we had some studies produced by the same group of authors, we screened them in-depth and kept an extended version of the work. In addition, seven papers were excluded from review as we could not access full texts. As a result, we were left with 288 papers for the final analysis - annotation.

### 3.3 Coding Framework

To identify the categories for data analysis, we integrated and adapted the HRI taxonomies from previous studies (Yanco and Drury, 2004; Bethel and Murphy, 2010; Salter et al., 2010; Tsiakas et al., 2018; Baraka et al., 2020; Onnasch and Roesler, 2020) and annotated the papers by the predefined categories. We describe below the different annotations used. These were used to produce quantitative analysis and to identify trends.

#### 3.3.1 Geographical Distribution

This information is not easy to infer from the publication; we chose to manually extract this information by checking the author’s affiliation and address, and country on the paper. While not perfect, we believe that it should give us a reasonable estimation of the country where the research was conducted for most articles.

#### 3.3.2 Type of Contribution

The field of HRI is very interdisciplinary. Inspired by the research themes of the ACM/IEEE HRI conference^9^, we chose to annotate the type of contribution according to four themes:

• *User studies* provide rigorous data on and analysis of HRI in the laboratory or in-the-field settings. They also should present sound methodology (quantitative, qualitative, or both) and accurate analyses that result in novel insights and acknowledge the limitations and relevance of the methods. Papers that presented an empirical evaluation with human participants were annotated as a user study.
• *Technical* papers are motivated to improve robot’s behaviors for the purposes of better interaction and collaboration with humans. The question of how technology advances HRI is key to these studies. They should include novel robot system algorithms, software development technologies, and computational advancements in support of HRI.
• *Design* contributions target research that takes a design-centric approach to HRI. They usually discuss the design of new robot morphologies and characteristics, behavior patterns, and interaction methods and scenarios, among many others. They should demonstrate essential or better interaction experiences or behaviors for robots.
• *Theory and methods* aim at unpacking fundamental HRI principles that include interaction patterns, theoretical concepts, updated interpretations of existing results, or new evaluation methodologies. Such papers might originate from original studies and existing research and methods or may take solely theoretical or philosophical perspectives.

#### 3.3.3 Research Contributions

Looking at all the papers in the selection, we identified the main research objective (e.g., facial recognition, non-verbal communication, programming framework) for each paper. We then grouped these objectives into several classes of contributions: robot perception and recognition (emotion, facial, object, body, sound, speech, gesture, color, gender, text), robot’s communication (verbal, non-verbal), reinforcement learning, and cognitive architecture. Imitation and display of emotions are separated from non-verbal communication due to a greater focus on them in observed studies. Apart from them, we included kinesthetic learning, physical exercises, taking an object, walking, and moving body parts. Some studies are both technical and user study, and there is more than one contribution example per paper.

#### 3.3.4 Application Field

Baraka et al. (2020) provided a cross-sectional snapshot of key application areas for social robots, and, intuitively, robots are used in more than one field. Our categories included: autism therapy, education, elderly care, healthcare, learning disabilities, public service, entertainment, art, sport, and generic.

#### 3.3.5 Human-Robot Ratio

Goodrich and Schultz (2007) considered that the ratio of people to robots directly influences the human-robot interaction. This taxonomy classification defines the number of a robot(s) and a participant(s).

#### 3.3.6 Participant’s and Robot’s Role

Goodrich and Schultz (2007) identified HRI roles, which were adopted by other researchers (Yanco and Drury, 2004; Tsiakas et al., 2018; Onnasch and Roesler, 2020). Based on their classification, 12 distinct participant’s roles and eight robot’s roles were defined. The description of each role is shown in Table 2.

**TABLE 2:** The description of roles for participant and robot.

	Role	Description
Participant	peer	interacts with a robot to achieve a shared goal
	coperator	works with a robot to fulfil a shared goal and does not directly depend on a robot
	collaborator	works as a teammate together for joint task completion
	learner	learns something from a robot
	imitator	imitates a robot’s gestures or action
	interviewee	answers to the questions from a robot
	mentor	takes on a leadership or teaching role
	supervisor	monitors a robot and gives instructions on how to perform the task
	operator	is aware of where and what a robot is doing
	mechanic	works with robotic software or hardware and controls the physical setting
	information consumer	does not necessarily interact with a robot, but uses information that comes from it
	bystander	does not interact with a robot but shares the same space
Robot	peer	acts as a friend to achieve a common interaction goal
	learner	acquires new skills or behaviors from humans
	tutor	supports learning by being in a teaching position
	mediator	enables an interaction between two or more people, so that they can engage through a robot
	assistant	performs actions alongside humans (e.g. a teaching assistant)
	interviewer	asks questions
	demonstrator	shows model behaviors or actions
	testbed platform	validates or tests theories and algorithms in an experiment

#### 3.3.7 Input and Output Channels

Onnasch and Roesler (2020) presented a taxonomy category which is named as the communication channels, split into input and output to highlight the human-robot interaction. Input describes how the robot “perceives” information coming from the human. Humans may provide information either using an electronic (e.g., remote control through the device), a mechanical (e.g., robot’s kinematic movement), an acoustic (e.g., commands), or an optical channel (e.g., gesture control). In turn, the robot’s output can be transmitted to humans through tactile communication (e.g., haptics), an acoustic (e.g., sounds), and a visual channel (e.g., eye movements). In the current study, the major distinction is that we view the input as any information coming from the environment (e.g., camera), while the output is what the robot produces through its channels (e.g., speech).

#### 3.3.8 Robot’s Autonomy Levels

According to Salter et al. (2010), the robot’s level of autonomy is defined as shown in Table 3.

**TABLE 3:** The level of robot autonomy.

Level	Description
Wizard of Oz (Woz)	the robot is controlled by a human in the non-collocated environment where the robot is present
Autonomous	the robot acts based on its input without any external human control during decision-making
Combination	the robot integrates different levels of autonomy (e.g. controlled fixed command patterns)
Scripted/fixed	the robot follows scripted spatio-temporal command patterns, despite the external factors
Teleoperation	the robot is controlled by a human present in the same environment as the robot is

#### 3.3.9 Experimental Methodology

Based on the classification proposed by Bethel and Murphy (2010), a study design is grouped into three categories:

• Within-subjects design - each participant undergoes the same experimental condition and is exposed to all levels of the independent variables.
• Between-subjects design - participants are exposed to different groups where each group experiences different conditions.
• Mixed-model factorial design - the use of both between-subjects and within-subjects design components.

#### 3.3.10 Duration of Interaction

Human-robot interaction studies can be grouped on the basis of the duration of interaction, which means the certain period of time when the human interacts with the robot (Baraka et al., 2020). Albeit it is challenging to define set boundaries between interaction times, we decided to follow the proposed duration looking at the number of sessions. We annotated according to the following categories: short-term (single or few interactions), medium-term (several days or weeks), long-term (extended period).

## 4 Quantitative Results

We propose to address our research questions with quantitative analysis to look at research trends over the years and the different categories identified above. All the graphs were generated using Altair, which is the declarative statistical visualization library for Python (VanderPlas et al., 2018).

### 4.1 Geographical Distribution

**FIGURE 2:**
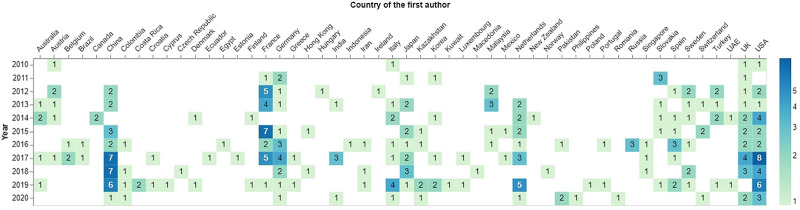
Number of publication per country.

Figure 2 shows the frequency of publications across countries and per year. Earlier works that date back to 2010 were produced in anglophone countries such as the US and UK and European countries including Austria and Italy. France being the NAO’s homeland, it also figures among the countries reporting a lot of research works. From the figure, it is apparent that the (predominantly) English-speaking world continues to dominate the HRI research with NAO. When compared to other parts of Europe, Nordic countries and Eastern Europe are substantially underrepresented. Notably, NAO has been used regularly in economically wealthy Asian countries such as China and Japan. Over the years, the largest number of papers were published by researchers from the USA (*N* = 33), China (*N* = 30), and France (*N* = 25). These results may serve as an example of widening digital inequity between countries with different economies.

Having said that, it is interesting to note that NAO was used quite broadly around the globe. Evidently, increasing the number of languages supported by the robot as shown in Table 1 has been an important factor in the integration of the robot. The language options for its text-to-speech API covering 20 languages can explain this broad use. We also can note that this multilingualism supports cross-cultural reproductibilty of previous studies and theories that were tested with NAO.

### 4.2 Research Contributions

**FIGURE 3:**
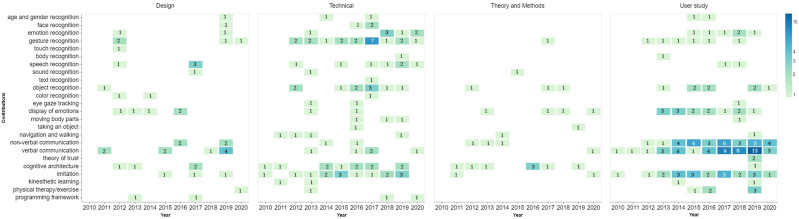
Contributions made over the years grouped by each study type.

Figure 3 demonstrates research topics that were identified as the papers’ main contributions. We group them by paper type and show their frequencies over the years. As of 2010, earlier contributions represent verbal communication, cognitive architecture, and imitation in technical and user studies. We cannot observe straightforward trends in design, theory and methods, but verbal communication and cognitive architecture seem to have a proper representation as common contribution topics. Our analysis shows that verbal (e.g., dialogues) and non-verbal communication (e.g., joint attention) were the most common contributions among user studies published in 2019. Gesture recognition was generally observed to be a popular contribution topic in technical papers, especially in 2017. Color, face, touch, and sound recognition were among the least popular topics for contributions, probably because of NAO’s limited perception abilities. It is important to note that some technical contributions (e.g., emotion recognition) are present within user studies or theory and method groups due to a single paper having several contributions. The more consistent distribution of design, theory and methods, and technical contributions, and the increasing rate of user studies through the years shows how the first three have contributed to the integration and testing of the robot in various domains through user studies.

### 4.3 Application Fields

**FIGURE 4:**
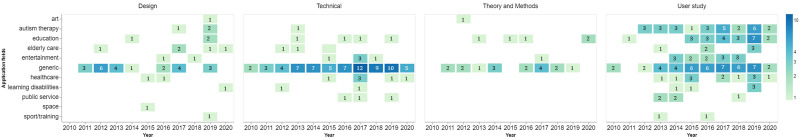
Application fields grouped by each study type over the years.

The applications contexts of NAO are displayed in Figure 4. Evidently, generic fields are prevalent across all types of studies. This hints on how the community has been keen on developing the generic technology with the NAO robot with the goal of integrating it in various applications. Which, in turn can contribute to integrating the robot not only in the research domain but also in the real-world applications. Furthermore, this means that NAO is being used for non-specific purposes such as addressing general hypotheses and technical questions, as can be seen from the share of technical studies. In user studies, the use of NAO has expanded stably in healthcare, autism therapy, and education since 2015. We separated studies on autism therapy from healthcare as this context is receiving a growing attention within HRI. Some unusual application areas are space (helping astronauts in space flight-operations in Sorce et al. (2015), art (drawing on canvas in Gurpinar et al. (2012) and performing in theatrical play in Petrović et al. (2019).

### 4.4 Human-Robot Ratio

**FIGURE 5:**
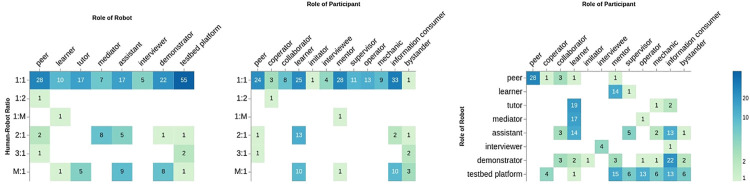
Human-robot ratio per role of robot and participant, and co-occurrence of robot and participant role **(right)**.

Figure 5 displays the ratio of participants to robots for various kinds of robot and participants’ roles. The vast majority of studies used one-to-one interaction with the roles of the robot as a testbed platform (*N* = 55) and the role of the human as an information consumer (*N* = 33). In a dyadic interaction, the robot quite often played a role of a peer (*N* = 28), demonstrator (*N* = 22), tutor (*N* = 17), assistant (*N* = 17), followed by learner (*N* = 10), mediator (*N* = 7) and an interviewer (*N* = 5). Participants often played the role of a mentor (*N* = 28), learner (*N* = 25), and peer (*N* = 24).

The ratio of many participants to a robot (*M*: 1) comes second with the robot roles of assistant (*N* = 9) and demonstrator (*N* = 8). In this context, humans were introduced as information consumers and learners in 10 studies for each. Triadic interaction was common among mediator and assistant robot roles and human learners (*N* = 13). Only a few studies had the ratio of 3 : 1 with no obvious trends.

The first trend shows that the majority of studies were carried out using dyadic interactions. The limited number of studies with two robots or more can imply either on the difficulties of developing multi-robot interactions or lack of interest in the community. Furthermore, while there are quite a few number of studies on triadic interactions with two humans and one robot, they are still limited to specific types of interaction where the human is a learner or an information consumer. On the other hand, after dyadic interactions, the most number of publications were carried out with one robot to more than five human ratio, with the robot being a demonstrator, assistant, or tutor. The analyses shows the number of studies using such dynamic has increased over the years.

### 4.5 Human-Robot Roles

In Figure 5 (right), we also demonstrate robot-participant interaction roles. It becomes clear that NAO generally plays collaborative and mediating roles. Our analysis shows that the most common HRI roles with NAO have been: peer-to-peer (*N* = 28) and demonstrator-to-information consumer (*N* = 22). When the human was in the learner’s role, a robot was most frequently in the role of either a tutor (*N* = 19), mediator (*N* = 17) or an assistant (*N* = 14). Our analysis presents the interviewee-interviewer dyad as an exceptional and interesting case in HRI. The examples of peer interaction include learning a language (Kim et al., 2019), playing a game (Hirose et al., 2015), and working as a team (Mubin et al., 2014). NAO as demonstrator or presenter performs actions in front of the participants (Krogsager et al., 2014; Wang et al., 2014; Kaptein et al., 2017). Learner-tutor interaction occurs in educational settings where NAO is engaged with language teaching (Kose et al., 2014; de Wit et al., 2020) and providing emotional support (Miskam et al., 2015; Cuadrado et al., 2016). NAO as a mediator predominantly helps children with autism or learning disabilities to scaffold social and communication skills (Shamsuddin et al., 2012; Huskens et al., 2016; Ioannou and Andreva, 2019). A further analyses of the dynamics between the role of the robot versus participant show some pairs of roles appear more than others. For example, there is a consistent use of robot as a testbed platform with human acquiring various roles such as mentor, mechanic, or information consumer. On the other hand, we can see lots of studies with human as a learner where the robot might have been a tutor, mediator, or assistant. It is also important to mention, some dynamics such as peer/peer or learner/tutor are more common in education and autism therapy.

### 4.6 Number of Participants and Age Groups

**FIGURE 6:**
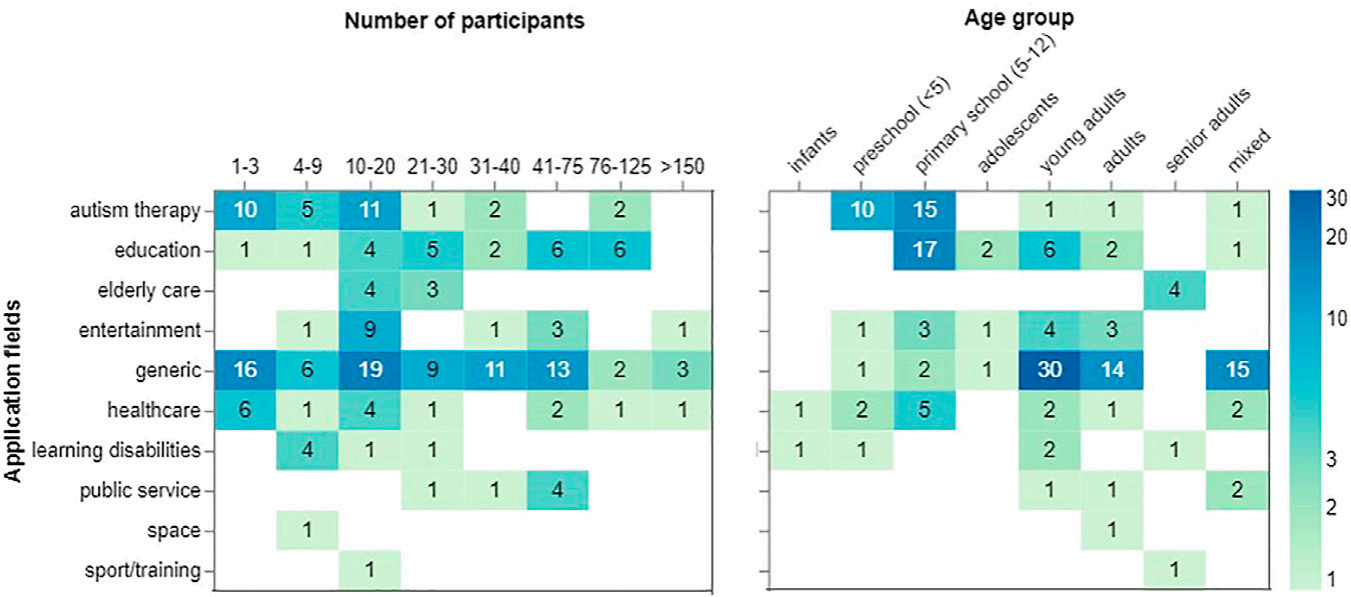
Number and age group of participants per application field.

Figure 6 juxtaposes how often the user studies had various ranges of participants. For the most part, the number of participants ranges from 1 to 20 people, having the greatest number in the range “10–20.” A smaller number of participants (up to three people) is mostly used for autism therapy, generic, and healthcare applications. A fair amount of generic studies recruited a large number of participants ranging from 30 to 75. Interestingly, studies conducted for education recruited the biggest number of participants that can go up to 125 people. There were a few entertainment, generic, and healthcare studies that had more than 150 participants.

Figure 6 (right) demonstrates the total number of studies that had various age groups for each application field. Children at preschools and primary schools participate in studies that focus on education (*N* = 17) and autism therapy (*N* = 25). Generic fields work with much older age groups since the studies are typically conducted with university students or staff (e.g., (Stadler et al., 2014; Celiktutan and Gunes, 2015)). The figure also reveals that senior adults interact with NAO for elderly care and learning disabilities applications. Infants and adolescents are the least represented age groups.

Figure 6 (left) shows that some application types such as autism therapy and healthcare use a smaller number of participants per study ( <20 ). A quick look at the distribution of age groups in autism therapy showed more focus on preschool and primary school aged children. This can explain the possible difficulties in recruiting participants for autism therapy studies which can be one of the causes of small sample sizes. On the other hand, educational user studies tend to have a higher number of participants (between 20 and 125) with the age group distribution of primary school and young adults. One of the interesting trends is the higher population of young adults and adults in generic studies, which can be explained by the possible easier procedure to recruit them for user studies. Whereas, most studies with children and the elderly that might be harder to recruit are conducted for specific applications such as autism therapy, education, and elderly care.

### 4.7 Input and Output Data

**FIGURE 7:**
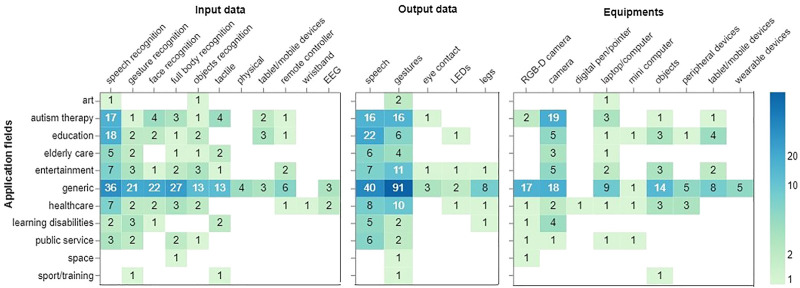
Input data **(left),** output data **(middle),** equipment **(right)** for each application field.

Figure 7 provides the frequency of input and output data throughout the application fields. Primarily, generic studies deployed speech (*N* = 36), full-body (*N* = 27), face (*N* = 22), and gestures (*N* = 21) as an input data for recognition. Interestingly, tactile input is mostly used within generic types of applications, with a few studies in autism therapy, elderly care, and learning disabilities. Tablet and mobile devices were mostly used for autism therapy, education, and generic fields. The least popular types of input data come from wristbands and electroencephalography (EEG). This might be due to the intrusive features of most wearables.

In line with these results, NAO’s output data is mostly speech and gestures in generic fields, autism therapy, and education. Eye contact and LEDs were comparatively less used by the robot.

Considering the various types of studies conducted with the NAO robot, we also looked at the type of equipment used alongside the robot. Figure 7 shows the input data (left), output data (middle), and equipment (right) used over all application fields. Speech recognition dominates the type of input data, which has been used on almost all of the application types, and it is proceeded by gesture, face, body, and object recognition. It is notable that apart from generic applications, higher use of speech recognition can be seen in autism therapy and education. Considering the target age groups for these applications, this calls for more attention in developing speech recognition technologies for children. As for the output data, 7 (middle), most applications seem to have utilized the robot’s speech and gestures. Autism therapy, entertainment, and healthcare had shown a higher tendency of using gestures in comparison to other applications.

### 4.8 Equipment

Figure 7 (right) also presents the use of different equipment that researchers make use of during their user studies. The most popular equipment are RGB-D cameras, ordinary cameras, and physical objects (e.g., a ball, geometric figures). Again generic studies employed these equipment more often than any other field. Tablet or mobile devices are commonly used in educational settings. Some examples of wearable devices are a helmet with electrodes (Gomilko et al., 2016), pluggable eyebrows to express emotions (De Beir et al., 2016) and peripheral devices such as a microphone, keyboard, and LCD monitor to show visual stimuli (Hu et al., 2016). Looking at the additional equipment used with the NAO robot, one notable trend is the additional usage of the camera and RGB-D camera alongside the NAO robot. While the camera might have been used to provide additional data from different angles to analyze the participant or the interaction, the use of RGB-D cameras, specifically in case of its placement from the robot’s point of view, can hint on the possible use cases of adding such a gadget to the robot, even as a supplementary item. Other equipment frequently used are laptop/computer and objects which depending on the activity, can add more interaction dimensions and modalities to the robot.

### 4.9 Robot’s Autonomy

**FIGURE 8:**
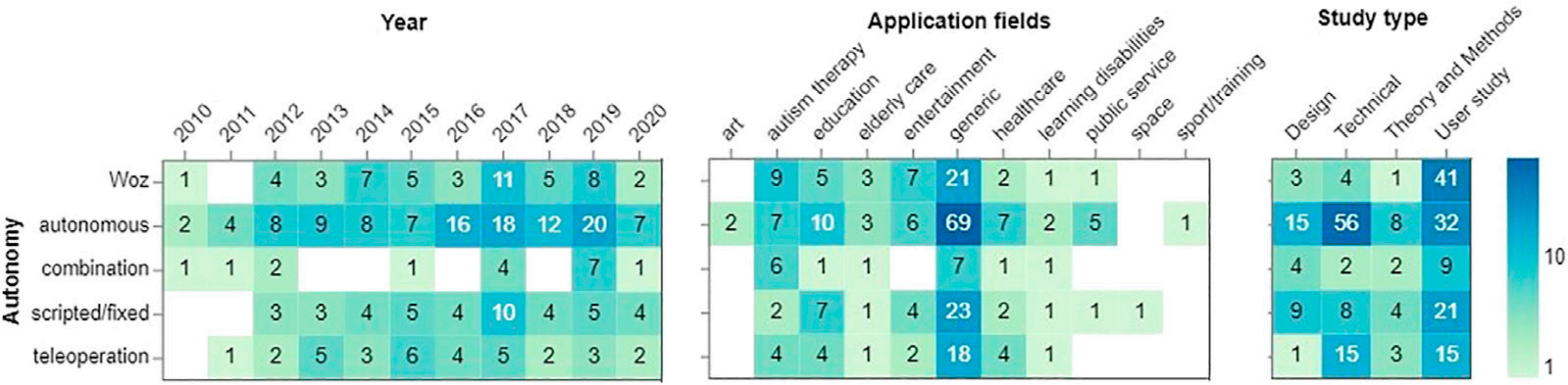
Robot’s autonomy per year and application fields.

Figure 8 illustrates the levels of robot autonomy by year and application fields. We observe clear trends that NAO is becoming more autonomous in recent years, with a significant increase from 2016 to 2019. Wizard of Oz is the second most widely chosen control type that has been evenly spread across the given years, except for 2011. Only generic fields appear to use all levels of robot autonomy, as a rule, autonomous mode, when compared to other fields. Novel application fields (space, art, and sports) constitute the least share in robot autonomy. Essentially, we can also report that technical studies use autonomous mode, while user studies give preference to the WoZ setting. In fact, a robot’s autonomy greatly varies in user studies as the modes are divided proportionately. The combination mode appears to be unpopular across all study types.

As we know NAO robot comes with NAOqi, a visual interface called Choregraphe and can be programmed using ROS. These all give the user plenty of opportunities to develop behaviors and interactions with the robot. As a result, in Figure 8, we looked at the distribution of the robot’s autonomy over the years (left), per application fields (middle), and study types (right). One noteworthy trend is the increasing rate of studies with the fully autonomous robot through the years, more specifically in 2016, 2017, and 2019. This can hint on how the technical developments and increasing interest in using the robot have contributed to the more autonomous deployment of the robot. After generic applications, education, autism therapy, and healthcare had the highest population in using NAO robot autonomously. It is worth mentioning that more studies in autism therapy have used Wizard of Oz than fully autonomous, which can also be explained by the restriction associated with running studies in this field. Looking at the autonomy versus study types (right), it can be seen that Wizard of Oz autonomy was more popular in user studies which can be explained by considering the difficulties of deploying a fully autonomous robot to interact with users. On the other hand, the fully autonomous robot has been used more in technical studies, then in user studies, and finally in design studies.

### 4.10 Experimental Methodology

**FIGURE 9:**
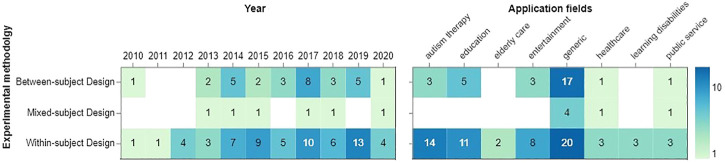
Experimental methodology per year **(left)** and application fields **(right).**

Figure 9 illustrates the frequency of using three types of experimental methodology across years and application fields. Seemingly, a within-subject design was commonplace from 2012 onwards. It reached the maximum number of publications (*N* = 13) in 2019, and the three most common fields of its use are generic, autism therapy, and education. Generic fields again lead the field by deploying both within- and between-subject design. Studies on autism therapy and education adopt the two designs. Studies in healthcare and public service choose between-subjects design rarely than any other field.

We have also analyzed the experimental methodologies used in user studies, both through the years and based on application fields as shown in Figure 9. As seen from the figure, the use of within-subject experimental design has increased through the years, and it is generally used more than between-subject and mixed designs. And among application fields, autism therapy, education, and entertainment were more prone to using within-subject designs. Apart from methodology, we also looked at experiment duration, as categorised in short, medium, and long-terms.

### 4.11 Duration of Experiment and Sessions

**FIGURE 10:**
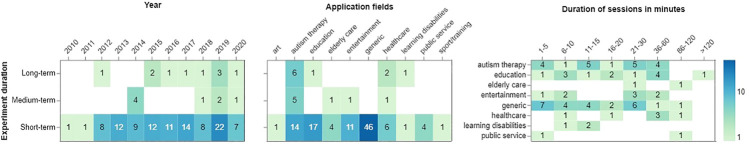
Timespan of the experiment per year **(left)** and application fields **(middle)** and duration of sessions in application field **(right).**

Figure 10 shows how long human-robot interaction lasts across years and fields. We see clearly that the majority of studies are short-term, specifically between 2015 and 2019. This obvious trend is explained by the prevalence of generic fields. Medium-term and long-term studies were scarce before 2014, but their numbers relatively increased by the mid-2010s. Only several studies that focus on autism therapy with NAO used a long-term approach. Despite no explicit trends, we can observe that the interaction up to 30 min is more common compared to other periods of time, mostly in generic and autism studies. Considerably, a few studies (*N* = 5) lasted for more than 60 min.

Figure 10 (left) shows the majority of the studies have been conducted on a short-term basis, and as the number of studies increased through the years the number of short-term studies has increased as well. There is no visible trend of increasing long-term studies at least with the NAO robot which can be thought provoking and worth understanding its underlying causes. As human-robot interaction field is thriving to understand the dynamics between human and the robot, we need more long-term studies to be able to show how the robots can integrate into our lives and society. Looking at Figure 10 (middle), we can see all generic studies have been conducted with short-term duration. It is intuitive to conduct a short-term study when developing or testing technology for generic purposes and invest more in running long-term studies with the specific application in mind. For example, studies on autism therapy and healthcare were more likely to have medium and long-term duration than the rest of the applications. The Figure 10 (right) shows a quick overview of the duration of the sessions in minutes. The duration of sessions is a function of the application and the interaction; hence we cannot observe a particular trend. However, it is interesting to see that people have been participating in experiments with NAO robots that had lasted up to 120 min. In general, the more we are trying to integrate robots into society and move them from research labs into the wild, we might need to run more long-term studies.

### 4.12 Concluding Remarks

The noteworthy findings that emerge from this quantitative data are:

• While studies with NAO have been produced all over the world, the great majority of studies are published by researchers in the Global North, particularly in the U.S and Western Europe.
• NAO has been used for generic purposes, yet it appears to gain traction in autism studies and education since 2015.
• Despite physical limitations, speech, and gestures are the main communication channels for NAO to interact with the environment. The lack of accurate speech recognition and natural behaviours such as emotions and spontaneity causes mixed feelings about its social use.
• Although efforts have been made to allow NAO to function autonomously in generic fields, it still depends on human control and supervision when interacting with the end-users (such as children).
• Humans from different age groups can interact with NAO, depending on the variation in contexts of use. Therapeutic and educational studies recruit primary age children, while generic studies mix up all available age groups. Dyadic interaction prevails significantly.
• The most recurrent robot roles for NAO are found to be peer, demonstrator, tutor, and mediator.
• The studies with NAO are predominantly short-term and may last for approximately 15–30 min.
• The available studies apply within-subject design more often than between-subject or mixed-subject. This is indicative of its relatively easier process as the number of participants can be smaller.

## 5 Qualitative Results

We also conducted a qualitative narrative review to discuss the primary research focus reported in the papers present in our collection. This section is concluded with the key findings that emerge from the literature.

### 5.1 The Human Perception of NAO

The way robots interact with humans is critical to evaluate the overall quality of robot capabilities. HRI has drawn great attention in studying how humans perceive robots in terms of their appearance, task performance, and communication skills, among many other robot features. User perceptions and experiences with NAO vary from one context to another as well as between user populations, including children, parents, teachers, and experts. Due to its small size, NAO is predominantly used in the child-robot interaction scenarios (*see* Figure 6), with some exceptions, in elderly care. Nevertheless, the majority of users perceive NAO as a friendly and sociable robot (Hernandez-Cedeño et al., 2019; Turp et al., 2019). There were also reports of mixed feelings about the robot, considering its physical and technical limitations (Cruz Maya et al., 2015; Sarabia et al., 2018). Additionally, the human-like appearance and non-judgemental characteristics of NAO are highly appreciated by users (Henkel et al., 2019; Olde Keizer et al., 2019). Users would like NAO to be more emotionally expressive, responsive, and have a natural voice and gesturing (Anastasiou et al., 2013; Ahmad et al., 2017). Authors used a variety of questionnaires to evaluate NAO’s characteristics and performance based on: anthropomorphism (Zlotowski et al., 2014; Kraus et al., 2016), user experience (Alenljung et al., 2018; Olde Keizer et al., 2019), user acceptability (Ahmad et al., 2017), robot personality (Liles and Beer, 2015; Peters et al., 2017; Kraus et al., 2018), robot behaviors (Pan et al., 2013; Njeri et al., 2016; Rossi et al., 2019), user expectations and evaluation (Anastasiou et al., 2013; Henkel et al., 2019), and perceived trustworthiness (Jessup et al., 2019). Table 4 presents common questionnaires that are used in evaluating human-oriented perception of NAO.

**TABLE 4:** Perception questionnaires commonly utilized in the reviewed studies.

Name	Author	Measurements	Item type
Godspeed Questionnaire Series (GQS)	Sturgeon et al. (2019)	anthropomorphism, animacy, likeability, perceived intelligence, and perceived safety	5-item with 5-point Likert scales
Unified Theory of Acceptance and Use of Technology (UTAUT)	Sinnema and Alimardani. (2019)	anxiety, attitude towards technology, perceived enjoyment, perceived sociability, perceived usefulness, social influence, and trust	7-item with 5-point Likert scales
Negative Attitude Toward Robots Scale (NARS)	Mirnig et al. (2017)	attitude toward interaction with robots, social influence of robots, and emotions in interaction with robots (e.g. I would feel relaxed talking with robots)	10-item with 5-point Likert scales
System Usability Scale (SUS)	Olde Keizer et al. (2019)	attitude towards usability (e.g. “I thought the system was easy to use,” “I felt very confident using the system”)	10-item with 5-point Likert scales
Individual Differences in Anthropomorphism Questionnaire (IDAQ)	Zlotowski et al. (2014)	anthropomorphic (“durable,” “useful,” “good-looking,” “active” and “lethargic”) and nonanthropomorphic traits (intentions, emotions, consciousness, free will, mind)	30-item with 10-point Likert scales
Complacency-Potential Rating Scale (CPRS)	Zlotowski et al. (2014)	attitudes towards automation (confidence-related, reliance-related, trust-related, and safety-related complacency	20-item with 5-point Likert scales
Propensity to Trust Technology (PTT)	Jessup et al. (2019)	attitudes towards technology and collaboration with technology (e.g. “Generally, I trust technology”; “Technology helps me solve many problems”)	6-item 5-point Likert scales
Robot Interactive Experiences Questionnaire	Mubin et al. (2014)	attitudes towards engagement and social interaction (e.g. alive, friendly, social)	8-item with 7-point Likert scales

When touching the robot, positive experiences with NAO were characterized as fun and engaging, while negative experiences were described to be odd and unsafe due to its small size and hard surface (Alenljung et al., 2018). Comparing low and high interactivity, Tozadore et al. (2017) found that children enjoy their experience with the high interactive NAO that use a warm greeting and recognizes their names. When compared to the virtual agent, users still favored NAO to be engaging and responsive (Artstein et al., 2017). Both teachers and students were comfortable with the use of NAO, yet they emphasised the need for facial expressions in NAO (Ahmad et al., 2017). Gender might influence how robots are perceived. For example, users found a male NAO more trustworthy and competent than a female one, which was only rated as likable (Kraus et al., 2018). In another study, children at different developmental stages had varying preferences towards NAO’s gender: younger children (5–8 years old) wanted a robot that matched their own gender, while older children (9–12 years old) did not have such gender-driven preferences (Sandygulova and O’Hare, 2015).

### 5.2 Verbal Communication With NAO

NAO can also be presented as a conversational companion to assist people with daily tasks, serving as language tutors in autism therapy (Fuglerud and Solheim, 2018), facilitators in speech therapy for hearing-impaired children (Ioannou and Andreva, 2019), and peers for self-disclosure among people with visual impairments and intellectual disabilities (Eyssel et al., 2017; Groot et al., 2019). Interestingly, NAO can also act as a storyteller that sustains children’s attention due to novelty and gesture frequency, while human storytellers may become fatigued to deliver the story (Wu et al., 2018; Ruffin et al., 2020). These studies also suggest that timely feedback during human-robot communication has been regarded as a success factor contributing to the quality of interaction. By presenting NAO as a talking partner, Omokawa et al. (2019) distinguished between two dialog types for verbal interaction: query type is a question-and-answer format, and phatic type is a casual format that involves small talk and/or personal feelings (e.g. acceptance). As the authors noted, human utterances are hardly recognized in the latter dialog type due to short words that probably express emotions. In Da Silva et al. (2018), NAO as a motivational interviewer also enabled verbal communication with humans, yet its lack of personalization was disliked by many participants (e.g. repeating the question an user had already answered). Recently, Graña and Triguero (2019) proposed a spoken dialogue system for the NAO to learn to answer autonomously based on human input^10^. For human-friendly communication, Manome et al. (2020) developed a machine translation system in which the NAO correctly speaks Japanese words that were converted into morphemes to enable easier pronunciation. The examples above indicate great prospects for the NAO to improve its verbal skills that are necessary for natural communication with humans.

### 5.3 Non-verbal Communication With NAO

In the same way, NAO’s non-verbal social cues play an important role during human-robot interaction. Non-verbal communication happens in many instances that help facilitate joint attention, turn-taking, shared attention during HRI. Although NAO lacks orientable eyes, which may be counted as a serious limitation, results indicate that head rotations typically help imitate eye contact (Cuijpers and van der Pol, 2013). For instance, NAO can serve the needs of children with autism who often find eye contact with other people uncomfortable and therefore try to avoid it. Additionally, different visual stimuli such as changing eye colour cyclically and blink by NAO were added to encourage eye contact with children (Ismail et al., 2012; Ali et al., 2020). Eye contact and turn-taking usually fit together, for example, when children kick the ball and wait for NAO to kick it back (Tariq et al., 2016). Gazing behavior, however, is the important sign of communication because it allows people to infer engagement and intent. NAO gazes over objects of its attention and points to them to elicit joint attention (Anzalone et al., 2015). These examples demonstrate the extent to which the child’s eye movements would be responsive when NAO directs its attention to other objects. NAO was able to perform Turkish Sign Language gestures (Kose et al., 2014). In a buyer-seller negotiating, a human-robot handshake prior to negotiation may benefit both sides to reach a more positive outcome (Bevan and Stanton Fraser, 2015).

### 5.4 NAO as a Support for Affective Computing Research

NAO cannot directly express emotions through facial expressions, yet it can perform acoustic and physical expression of emotions. It is viewed as one of the limitations in its design. Most research studies proposed to express emotions through utterances (De Beir et al., 2016), gestures (Beck et al., 2012; Erden, 2013; Miskam et al., 2013; Rudovic et al., 2017), or both (Aly and Tapus, 2013; Tielman et al., 2014; Miskam et al., 2015). A few others attempted to use innovative ways such as eyebrows showing emotions (De Beir et al., 2016) and motion planning for four emotional patterns (Wei and Zhao, 2016).

Beck et al. (2012) designed six key poses that were implemented on the NAO to display emotions such as anger, pride, sadness, happiness, fear, and excitement, as captured by a motion camera system. Similarly, Erden (2013) adapted emotional human postures to the robot that expressed anger, happiness, and sadness through 32 different postures for each emotion. Creatively, De Beir et al. (2016) used 3D-printed and wearable eyebrows that allow NAO to show anger or sadness while doing other tasks simultaneously. In a play scenario, NAO can also show excitement and enjoyment using matching phrases such as “I am really excited!,” “I enjoy playing with you!” while no emotional posture or saying is expressed in a boring or tiresome state (Franco, 2015). Miskam et al. (2015) proposed to use NAO for teaching emotions using LEDs, hand or body gestures to the children with autism, who then imitate the robot by repeating the emotions such as being happy or hungry. Rudovic et al. (2017) also used NAO for robot-assisted autism therapy, where children had to recognize different robot emotions as shown in emotion cards. Through Laban Movement Analysis (LMA), Wei and Zhao 2016) integrated four emotional patterns into robot behaviours using motion planning. Interestingly, (Manohar and Crandall, 2014) studied how novice people program robot’s behaviors to express emotions through recorded audios and gestures and then recognized them. The study found that non-verbal emotions were not easy to discern than those expressed *via* verbal channels. NAO can also recognize human emotions through speech cues and facial expressions. For instance, Bechade et al. (2015) proposed an emotion recognition game in which the robot had to recognize emotions through the speech of humans. Likewise, Diaz et al. (2018) implemented a text2emotion system that enables NAO to execute behaviors based on its ability to recognize audiovisual stimuli. Stojanovska et al. (2018) tested NAO’s emotion recognition rate by recruiting participants to act emotions in front of the robot. Lopez-Rincon (2019) enabled NAO to recognize human emotions based on their photos on the computer screen, from which NAO detected one-half of the face images (535/1192) from the Child Affective Facial Expression (CAFE) dataset. Roshdy et al. (2019) applied a human brain-based mapping system for emotion recognition through Emotiv headset, motivated by the mapping of the human brain activity into NAO. In general, the robot can express and recognize emotions successfully except if users are not good at displaying them.

### 5.5 NAO as a Tool for Therapy and Learning

Despite many generic use cases implemented for NAO, this robot is widely deployed within medical and educational institutions for use by children.

Learning by imitation refers to observing and performing a new behaviour by replicating the action of others. Within HRI, imitation is generally practiced with children with health problems (e.g., autism) because they have difficulties in motor and/or turn-taking skills. When children mirror robot gestures and other behaviours, they can improve social interaction skills. In this way, most human-robot interaction occurs in a playful environment, where children, robot, or both imitate. In Arent and Kruk-Lasocka (2019), NAO played two interactive games with children to improve their turn-taking skills through movement imitation. Arias-Aguilar et al. (2017) designed a child-robot interaction in which NAO proposes typically developing children to a “play” by imitating the same arms and legs movements that it makes itself. Chevalier et al. (2017) designed a playful task in which NAO performed several hand gestures in the background of music with the same duration and rhythm. Both the robot and children with ASD had to imitate each other’s arm movements, but children were a bit confused to initiate them. In Di Nuovo et al. (2020), NAO presented itself and engaged with the children by playing music and storytelling and then asked to imitate its dance movements. Greczek et al. (2014) developed a Copy-cat game played between an NAO robot and a child with ASD. In the game, the robot asks a child to mirror its pose, saying, “Can you copy me?.” In learning by imitation framework, some authors propose to use Dynamic Time Warping that observes joint angles trajectories instead of Hidden Markov Models (HMM) for time normalization (Thobbi and Sheng, 2010).

Meanwhile, some researchers (Cazzato et al., 2019) proposed a system where NAO recognizes the presence of a user in real-time and imitates the human’s head pose. To augment motor skills, NAO may encourage imitation learning (e.g., sit-to-stand) in children with cerebral palsy, despite its physical limitation to move naturally (Rahman et al., 2015). In Ros et al. (2014), NAO taught children dance moves while providing verbal support with music. Tapus et al. (2012) developed a motor imitation task in which NAO imitates gross arm movements of children with ASD in real-time. The results show a high variation in children’s reactions to the NAO, which means that not all children can benefit in the same way. For rehabilitation and prevention of scoliosis (abnormal curve of the backbone), Vircikova and Sincak (2013) presented NAO in hospital and school settings. The participating children imitated NAO’s motions accurately, which also increased their motivation to exercise more. Quite similarly, NAO, as a trainer, performed physical exercises with elderly people who tried to imitate movements (Werner et al., 2013). In this context, users had to imitate mood-modified NAO’s arm gestures in a game, after which the robot provided verbal feedback about user performance (e.g., “Yes, those were the right gestures” for a correct movement). Imitation is one of the important skills for individuals with developmental disorders who need to understand social cues from a young age. Therefore, research shows that NAO is able to facilitate initiation and turn-taking skills through imitation tasks or games.

NAO is generally welcomed by students who view this robot as a learning peer, a more knowledgeable tutor, or a less knowledgeable learner (Johal, 2020). Rosenberg-Kima et al. (2019) found that the physical presence of robots brought positive changes for university students because of the technical functionality, social, and psychological activity. Namely, students pointed out the benefits as follows: “accessible to multiple people,” “immediate feedback,” “he is not judgmental like human beings,” “pleasant and motivating.” Some research has targeted specific skills required for language learning: reading (Yadollahi et al., 2018), grammar (Belpaeme et al., 2018), handwriting (Hood et al., 2015), alphabet (Sandygulova et al., 2020) or vocabulary learning (Balkibekov et al., 2016). Other research demonstrated that learners cultivate favorable impressions toward robots as learning companions, and the child-robot interaction may lead to increased self-confidence (Hood et al., 2015) and better task performance requiring creativity and problem-solving. Other studies e.g., Vogt et al. (2019) explored long-term learning between NAO and children to better understand this type of HRI in a real-world environment.

### 5.6 Typical Comparisons in HRI Studies With NAO

To identify the robustness and applicability of the social robot, comparative studies have been undertaken in terms of interaction roles and behaviors. Comparison occurs not only between robots but also between participants and interaction types. The comparisons between humans include children vs. adults (Kaptein et al., 2017), expert vs. non-expert (Ansermin et al., 2017), autistic vs. typically developing children (Anzalone et al., 2015), programmer vs. non-programmer (Stadler et al., 2014), and people from different cultures (Rudovic et al., 2017; Shidujaman and Mi, 2018). This shows that different groups of humans may have different experiences with a social robot.

Bethel et al. (2013) compared a human interviewer and a robot interviewer to find out which of them impacts participants when presented misleading information. The results show that the misinformation effect was significant in the human interviewer condition than in the robot interviewer condition. The authors suggest that its TTS system caused the lack of speech comprehension, which results in issues with the robot’s understandability. In Henkel et al. (2019), participants found the robot interviewer as non-judgemental with whom they were likely to share secrets. In a language learning context, a human teacher and robot teacher performed sign language gestures in a real and virtual environment, addressing the embodiment effect (Kose et al., 2012). Tapus et al. (2012) explored whether children with autism engage more with a robot partner or a human partner during a movement imitation task, in which no significant differences were found. In performing physical exercises (Werner et al., 2013), users perceived NAO as less motivating than humans, but they also rated the robot as more motivating than a standard training plan they use regularly.

When exploring robot embodiment, most users perceive NAO better in terms of its engagement and social characteristics. Artstein et al. (2017) found that a physical robot was more preferred and engaging to participants when compared with a virtual agent, which in turn led to better memorization over a longer period. Bevan and Stanton Fraser (2015) were interested in comparing telepresent NAO against non-telepresent NAO when shaking hands with participants during negotiations, whereas Tozadore et al. (2017) evaluated controlling the robot autonomously and through WoZ. Both studies suggest that a robot’s presence did not affect the degree of trustworthiness and appraisal, and user enjoyment, but the perceived level of robot intelligence may decrease when people know about teleoperation. Some studies explored robot personality effect on interaction quality such as extroverted vs. introverted (Aly and Tapus, 2013; Celiktutan and Gunes, 2015), low interactive vs. high interactive (Tozadore et al., 2016; Horstmann and Krämer, 2020), active vs. passive (Mubin et al., 2014), affective vs. non-affective (Tielman et al., 2014), emotional vs. unemotional and high vs. low intelligence (Zlotowski et al., 2014), lack of ability vs. lack of effort (van der Woerdt and Haselager, 2019), and simulated vs. real robot (Riccio et al., 2016). The robot-to-robot interaction and comparisons were also carried out in different contexts. However, only some papers compared the efficacy and utility benefits of the robots, mainly using the other robot as an alternative to the NAO or vice versa. Although children prefer NAO, they find easier to understand the gestures of a taller R3 (Kose et al., 2014) and rate Baxter robot as more positive and acceptable than NAO (Cuan et al., 2018). NAO was reportedly used along with Aibo in gesture experiments (Andry et al., 2011), iCub in eliciting behaviors on humans (Anzalone et al., 2015), Wifibot to carry the NAO (Canal et al., 2016), Pepper in human head imitation (Cazzato et al., 2019), Turtelbot in providing elderly care (DiMaria et al., 2017), Robokind R25 in interviewing humans (Henkel et al., 2019), Reeti (Johal et al., 2014) in expressing different parenting styles, R3 (Kose et al., 2014) in performing sign language gestures, Palro and Gemini (Pan et al., 2013) in evaluating interaction styles, and PR2 in identifying preferred human-robot proxemics (Rajamohan et al., 2019).

### 5.7 Novel Developments in Human-NAO Interaction

NAO has been used for unique purposes, which paved the way for new developments in human-robot interaction. One of the limitations of NAO is linked to physical abilities. Therefore, researchers try to improve physical contact with humans based on sensory information coming from them. Technical studies demonstrate promising results in relation to kinesthetic teaching by humans (Cho and Jo, 2011; Stadler et al., 2014). For instance, Bellaccini et al. (2014) proposed manual guidance of NAO without force sensors to improve physical human-robot interaction (pHRI). In a quite similar way, Berger et al. (2013) introduced a machine learning approach that enables NAO to follow human guidance by identifying human forces during a joint transportation task. Cao et al. (2014, 2017) presented a novel collaborative behavior controller ROBEE that selects actions based on homeostatic drive theory for NAO to jointly perform a task with participants more autonomously. In other words, this controller allows NAO to be aware of users’ psychological (e.g., valence) and physical (e.g., thirst) needs. The brain-machine interface (BMI or BCI) has been commonplace in studies that address the problems of people with motor disabilities. Accordingly, some researchers proposed a novel BMI interface such as EOG/ERP hybrid human-machine interface (Ma et al., 2013), EEG-based recognition of imaginary movements of fingers (Stankevich and Sonkin, 2016) and Emotive EPOC (Gomilko et al., 2016) to control NAO behaviours through commands by translating human brain activity. These findings show that NAO’s limitations might be overcome by using more advanced deep learning solutions that enable the robot to function in natural environments.

### 5.8 From Close-Loop Systems Towards Real Autonomy

The realization of versatile and human-like intelligence and cognitive modules in robots remains a challenge for the HRI field. As shown by our analysis, all autonomous systems used in user studies were targeting a specific application field. Generic reasoning relates to developmental robotics that can include various theories and methods such as deep learning, sensorimotor information processing, metacognition, memory, and decision making (Miyazawa et al., 2019). Several studies proposed a cognitive architecture for NAO’s system design. For instance, Adam et al. (2016) presented Cognitive and Affective Interaction-Oriented architecture (CAIO) that allows NAO to perceive its environment multi-modally, to manipulate mental states, to respond emotionally, and to execute physical and verbal actions. Aly and Dugan (2018) proposed Experiential Robot Learning in which NAO must autonomously learn and gradually acquire knowledge and skills through experience in the real world, achieved through reinforcement learning. Dindo and Zambuto (2010) focused on a multi-instance learning algorithm for NAO to learn the word-to-meaning associations through visual perceptions. Andry et al. (2011) presented an artificial neural network control architecture that allows rhythm detection to build an internal reward for learning inspired by human behavior. It has implications on the quality of the interaction in which NAO is capable of predicting and following human actions. To endow NAO with more adaptive behaviours, Bertacchini et al. (2017) designed a cognitive architecture that consists of human identification, emotions and gestures recognition and exhibition, and speech sentiment analysis in customer-robot interaction. Using computational modeling, Cantucci and Falcone (2019) endowed NAO with social autonomy in which it serves the role of infoPoint assistant that helps users to find out the location of their point of interest (e.g., a restaurant) and how to get to the place. Quite similarly, through the Internet of Things framework (IoT), Mondal and Nandi (2017) created a customizable assistant by enabling NAO to perform daily tasks that its owner requests. To increase the emotional aspect of interaction, Chella et al. (2013) built the cognitive architecture of NAO based on perceptual, emotional, and behavioural data. Another attempt in this area is made by Ribeiro et al. (2016) that presented the Socially Expressive Robotics Architecture (SERA) ecosystem for NAO as an autonomous and emphatic robot tutor in teaching sustainable development. These multiple examples of cognitive architectures for NAO are important to enable human-like intelligence and develop more natural HRI. A more detailed overview of research on cognitive architectures can be found in Ye et al. (2018) and Kotseruba et al. (2020).

### 5.9 Concluding Remarks

NAO is a well-accepted social robot valued for its fun and enjoyable appearance. However, there were mixed feelings about its interaction capabilities, which manifest diverse individual preferences and perceptions. Its interactive abilities can be empowered when displaying and recognizing emotions. Commonly, its body language is a medium for expressing emotions. NAO can detect emotions from facial expressions, and therefore, there is an emotional contagion in which NAO adapts to a human’s emotional state or vice versa (Xu et al., 2015; Stojanovska et al., 2018). Users also want NAO to feel and show different kinds of emotions. For instance, students thought they wanted NAO to “feel life” and feel happiness and togetherness when interacting with them (Omokawa and Matsuura, 2018). As compared to the unemotional NAO, the emotional robot was considered more anthropomorphic, while its intelligence may not affect the perception of anthropomorphism (Zlotowski et al., 2014).

NAO is widely accepted as a socially assistive robot, which communicates with users socially rather than physically (Sarabia et al., 2018). A great body of research has used NAO as a mediator in autism therapy and other therapeutic interventions with older people. Using social robots can offer alternative or complementary ways to support traditional treatment. As a viable approach to autism treatment, robot-mediated autism intervention is designed to improve children’s verbal and non-verbal behaviours as well as social skills. Levels of autism are known to be the most defining factor that accounts for different social interaction experiences and engagement rates (Ahmad et al., 2017). So far, the autism studies with NAO found that it has a great potential in helping children with autism to maintain eye contact (Anzalone et al., 2015), prefer specific instructions over spontaneity (Arent and Kruk-Lasocka, 2019) and augment communication skills (Hamid et al., 2013). Some other therapies focus on physical therapy, for instance, to improve motor learning skills of children with cerebral palsy (Rahman et al., 2015; Buitrago et al., 2020). Children with motor disabilities may become motivated and encouraged to do imitation and motor learning tasks. In addition, hearing-impacted children’s sound detection improved over sessions, meaning that NAO can be used for auditory-verbal therapy (Ioannou and Andreva, 2019). Verbal communication with NAO has occurred in different learning and communication scenarios. Its speech is mainly based on scripted texts and therefore usually lacks personalized responses. Thus, autonomous and natural human-NAO verbal interaction is still at its infancy.

Users liked the robot’s nonjudgemental behavior (Da Silva et al., 2018), and they were more engaged when the robot asked for personal details than quiz-like questions (Eyssel et al., 2017). In particular, game-based relationship with the robot may result in more self-disclosure (Groot et al., 2019). Furthermore, NAO was seen as more trustworthy and persuasive when compared to a virtual agent in either voice or virtual embodiment (Artstein et al., 2017). This distinctive characteristic hints that NAO can deal with sensitive issues carefully without making people feel uncomfortable when revealing oneself.

It was found that robots playing games with humans have an entertainment value (Johnson et al., 2016). Especially, it holds true for young children since their learning is mainly based on free play activities than instructed guidance on task performance. For instance, users preferred the R3 robot for learning, while NAO was associated with play (Kose et al., 2014). In another study, NAO played a board game known as tic-tac-toe with users on a tablet and showed that its behaviors could be robust with the help of voice synthesis and recognition. A more active, interactive, and extrovert robot is preferred as a partner in meetings (Mubin et al., 2014). There was no significant difference in user enjoyment between the system conditions, but most children tend to favor autonomous robot (Tozadore et al., 2017). Learning with NAO is interesting for children, and the content of play may affect the result of the learning process. Character recognition also plays an important role, how NAO recognises the kids’ writing, and it can be spelled back towards them. In this case, the kids can learn how to pronounce English much better and learn the handwriting of the alphabet (Kim et al., 2019). The two-way communication is found to be effective since each child can respond to the questions from NAO (Miskam et al., 2013).

Personalization is a much-needed feature for all social robots, and NAO’s case is no exception. It is commonly regarded that the robot may become more effective and human-like when it is able to tailor to user’s needs and preferences and build a sustainable and long-term relationship. Personalized human-robot interactions are specifically suitable when robots interact with humans for longer periods (Irfan et al., 2019). In such context, robots may develop a certain kind of memory storage that allows them to remember and record all available information about people through continuous interaction with humans. Considering the variation in autism symptoms, there is a clear need for robot personalization in autism therapy (Fuglerud and Solheim, 2018). To illustrate, Greczek et al. (2014) emphasized that varied feedback may be more effective and less discouraging than descriptive feedback in an imitation game for children with autism. Also, Mirnig et al. (2011) found that human-robot interaction might be affected due to the provision or withholding of feedback. Users’ perception of the robots could be distinguished based on different interaction styles even when it is a short-lived encounter (Pan et al., 2013). We come back to this subject later in the paper.

Personal characteristics of NAO are essential as each human shows idiosyncratic preferences in behaviours. What is interesting is that both introverted and extroverted humans wanted to interact with the personality-matching robot (Aly and Tapus, 2013). This posits that the personality traits of the robot are a relatively significant factor in relation to its non-verbal behavior. Users prefer to have a robot partner that shares the same personality as in the human-human interaction. Not surprisingly, it is suggested that extroverted robots positively affect interaction flow (Celiktutan and Gunes, 2015).

Similar to a human-human relationship, it may not be realistic if the human-robot interaction imitates faultless and impeccable communication. In psychology, *the pratfall effect* explains that a mistake would increase the interpersonal appeal and make humans more likable (Aronson et al., 2014). In this regard, Mirnig et al. (2017) highlights that the same phenomenon can be applied to social robots. In their study, participants liked the faulty robot significantly better than the robot that interacted flawlessly. The timing of the errors might also play an important role. Much interestingly, Lucas et al. (2018) found that NAO having conversational errors during warm-up conversation may recover sooner. Nevertheless, some users may develop biases toward the robot to be faulty and have limited skills (Turp et al., 2019). Although an erroneous robot is generally under-studied, it certainly is one of the key areas to understand human-robot interaction in an unrestricted way.

Researchers have used external measurement devices such as RGB-D camera, eye tracker, motion detector, and many other tools for some decades. They make it possible to measure human features such as body postures, movement, speech, and gaze in a more accurate and reliable way. They can fill the gap in the robot’s capabilities in measuring a human’s input and engagement. In Craig et al. (2016), gaze tracking hardware is used to create gaze-based language command in order to facilitate the communication barriers between NAO and users. In another study, a speech recognition system called Cloud technology was used to assess the correct understanding of Chinese language words that were transmitted to NAO (Han et al., 2018). Other researchers use gesture recognition system based on external cameras (Ajili et al., 2017) and object detection algorithm to recognize the face from NAO’s main camera (Cheng et al., 2012; Cuijpers and van der Pol, 2013). These advancements are significant as service robots are becoming popular in our domestic and social lives. In some way, it would be innovative if these technologies could also evaluate the quality of human-robot interaction. For instance, there might be some level of subjectivity in coding behaviours, especially in autism therapy (Baraka et al., 2017).

Existing research studies found no conclusive evidence regarding the benefits of social robots over other technologies. NAO’s advantage over other technologies is still unclear as there are insufficient evidence for its benefit compared to tablets and computers. It might be intuitive to consider that users prefer to interact with a physical robot because of its animate and lively appearance. However, a user preference may depend on other factors, such as age and context. Notably, older adults who have serious underlying health issues may be inclined towards physical robots. For example, elderly people preferred robots over a tablet, despite technical limitations of the NAO (Olde Keizer et al., 2019). Furthermore, students perceived NAO as a sociable agent and favored it over other learning aids, e.g., a computer (Liles and Beer, 2015). Focusing on a language learning context, Zhexenova et al. (2020) reported that there is no significant difference in children’s perceptions of NAO’s effectiveness in comparison with a tablet and a human teacher. In the entertainment area, Wong et al. (2018) revealed that physically present NAO improved information dissemination and hence increased visibility of the advertised product.

## 6 Key Insights: Strengths and Limitations

Our overall impression of the current review demands a further reflection on how research could be conducted with a social robot NAO. Although some points may be generic, we believe the research-based insights will benefit researchers either working or intending to work with NAO.

### 6.1 Strengths

*NAO is commonly regarded as a widely used platform.* Researchers tend to skip the details of why they choose NAO over other social robots except acknowledging its wider use. The most straightforward reason is that NAO has become the standard platform for RoboCup, an international robotics competition, for over 10 years.

*NAO enjoys a great appeal from its end-users.* Its child-like and non-threatening appearance makes it appealing. In particular, children at younger ages appear to engage with NAO more successfully than those at later childhood stages. This idea is supported by the significant number of studies that have been conducted in the educational and therapeutic context.

*NAO is certainly not just an eye-catcher robot as its portability is highly appreciated by the researchers.* Its small size in addition to light weight is helpful for easy transportation in a standard car (e.g. a taxi) which makes *in the wild* research possible.

*NAO can be regarded as a plug-and-play robot due to its robust and easy setup characteristics.* This allows researchers to have a reliable platform for a real-world deployment as confirmed by numerous research works conducted in diverse settings, ranging from schools to hospitals.

*NAO is an affordable robot with a cost of around 6000 Euro* ^11^ *.* Although it might be more expensive in comparison to other smaller humanoid robots, NAO is one of the most complete humanoid robots on the market in terms of functional and technical abilities.

*NAO’s customizable features also meet the needs of multi-disciplinary researchers worldwide.* This is surely thanks to the multi-level programming framework proposed to researchers. While the block-based programming framework, Choregraphe, allows researchers from social sciences to easily implement novel behaviors, the C++/Python API allows engineers to develop novel technical contributions (i.e. computer vision, control, etc.) and deploy directly on the robot. The HRI field being so multi-disciplinary, its programming framework positively contributed to the success of the NAO platform.

*NAO is multimodal in both its input and output communication modalities.* It is relatively well equipped with internal sensors to perceive its environment as well as external actuators to perform verbal and non-verbal behaviors (e.g. body motion and LEDs).

*NAO can take on a unique social role of one’s learner.* NAO as an educational robot has assisted poorly performing children to engage in a learning activity by taking up a unique social role of their learner. This can positively affect meta-cognitive abilities such as increased self-confidence and problem-solving (Hood et al., 2015). Other notable examples include handwriting practicing, second language learning, and studying school subjects like mathematics and science classes. With remarkable achievements in education, NAO is not much used in traditional and formal learning settings and rather acts as a one-to-one tutor, peer, or a learner (Johal, 2020).

*NAO can bring cognitive and affective values when interacting with humans that have social and learning barriers.* Although the robot can not replace the key social actors such as therapists and teachers, it can make learning and therapy engaging and fun experience, while educators can focus on creative as well as differentiated teaching practices.

*NAO could be a great help for individuals who have less social experience and companionship in their life.* For instance, in treating dementia and for other elderly care therapies, it could be applied to assist in daily life by monitoring and reminding to take the pills and do physical exercises following a certain plan instructed by medical staff. NAO as a companion may enhance the quality of life that most people expect to enjoy in their later lives.

*Gendered stereotypes seem to persist in human-robot interaction.* Multiple research indicate that users may perceive the robot in different ways based on gender markers such as voice and verbal commands (Sandygulova and O’Hare, 2015; Jackson et al., 2020). To a great extent, NAO is among the genderless robots (Obaid et al., 2016) compared to other robots (e.g., Kaspar, Milo). Thus, research with less gendered robots is important to eliminate gendered attitudes towards feminine and masculine qualities, which appear to contribute to the interaction outcomes.

### 6.2 Weaknesses

*NAO has a low battery life and overheating issues* that make it less durable than other social robots (e.g., Pepper). Generally, it works for 60 min in active use and 90 min in normal use. These issues question its sustainability and long-term efficacy. As our review shows, the majority of experiments with NAO usually happen on a short-term basis lasting for no more than 30 min. For instance, some participants are concerned with the robot being not active and responsive as they expected it to be. With that in mind, the activities and experimental design need to be adjusted time-wise.

*Although NAO is relatively well equipped to perform near-human actions, it is quite often supported by input/output external devices such as high-quality or depth cameras and microphones.* While NAO has two internal cameras, the low resolution does not allow to perform complex vision recognition tasks. For example, the closer a person is, the better the robot detects facial expressions and other visual cues, while it cannot recognize people who are more than 2 m away (Bolotnikova et al., 2017). Oftentimes, the use of additional equipment such as touchscreens, tablets, or wearables can substitute for perceptual limitations (Johal, 2020).

*NAO can hardly function in a noisy environment and recognize human speech.* User’s age influences speech recognition as young children and older people have different speech characteristics and coherence (Vargas et al., 2021). In particular, it is not yet practicable for NAO to recognize children’s speech (Kennedy et al., 2017). Alternatively, researchers could use Google Cloud Speech recognition services that allow NAO understand different languages and optimize its workflow.

*Hard surfaces are needed for NAO’s movements and stable positioning.* Aldebran first designed NAO as bipedal robot to walk in open loop. Closed loop walk algorithm was adopted on NAO humanoids that became capable of omnidirectional walking (Gouaillier et al., 2010; Kasari et al., 2019). NAO has a particular way of walking, and while the robot can move freely on flat and hard surfaces, it lacks robustness on surfaces such as on carpets or rugs (Shamsuddin et al., 2011). For instance, RoboCup teams like Berlin United (previously NAO Team Humboldt) have long been exploring the robot’s ability to move and kick the soccer ball autonomously based on visual spatial perception^12^.

*Autonomy is undoubtedly the most demanding feature that most social robots lack.* NAO has been predominantly used in the Wizard of Oz approach, a frequently employed method; wherein the interaction is controlled remotely by human input along the autonomy spectrum (Riek, 2012). Scripted, although highly constrained interactions are also commonly used solutions.

## 7 Future Research With NAO

Our results allow us to make a number of recommendations for future research using NAO:

Data-driven behavior generation: While rule-based behaviour generation approaches perform well, they are often costly, time-consuming and bound up to expert knowledge. The cost of creating production rules and the need for manual configurations in order to generate complex and natural human behaviours put a limit to the complexity and diversity of generated behaviours. Thus, the development of data-driven behaviour generating systems using machine learning have to become the research focus as the actual human-human interaction data can provide a more human-like and multimodal behaviour generation (*see* Liu et al. (2021) for a review on gesture generation).

Long-term engagement: Although cross-sectional studies are commonplace due to different technological and methodological constraints, it is feasible to commit to long-term goals and test the efficacy of NAO and its capabilities. The user studies in robot-assisted educational and therapeutic settings need convincing evidence of the robot’s long-term efficacy, especially those working with underserved populations (Rakhymbayeva et al., 2021).

Multi-party interaction: It would be suitable to observe and refine NAO’s behaviors and its relationship with study participants in the presence of co-present others. One-on-one interaction has long been practiced, however, it is still unclear how NAO interacts with multiple participants. This type of interaction deserves much attention because it allows to maintain collaborative HRI. The robot’s mediating role is important to facilitate human relationships such as student-student, student-tutor, and child-parent. In addition, professionals from other fields such as psychology and education can also contribute to evaluating the quality of human-robot interaction. For instance, in an educational setting, teachers may assess the interaction outcomes based on rubrics and observation.

Natural communication: Social dialogues should be more uplifting and engaging using more affective reactions. They may be based on a real interaction scenario where different participants react in varying ways. Interaction roles might be specified in advance, or users may find out in the course of the dialogue. Open-ended interactions can be designed where the robot is faulty or make errors during the interaction from which they can recover during the session. However, it might be helpful to maintain a cooperative imagined contact relying on real-life scenario. Research shows that imagined contact may provide humans with a behavioral guide, which probably improves their feelings of self-efficacy and confidence in future interaction (Kuchenbrandt and Eyssel, 2012).

Personalization: One cannot fit all, especially when it comes to social interaction. For that reason, it seems that adaptation and personalization have been under investigated as the NAO robot was used across various populations and cultures without much changes. Interventions have to be aware of user demographics which is the most straightforward way to adapt the content by adding specific verbal and non-verbal cues. The decision over how much personalization to use has to derive from study focus and population, which is highly anticipated of any experiment. In the case of young children with autism, there is a strong need for customized robot behaviors, as these children show varying degrees of autism symptoms that develop individually. For this reason, the NAO can target different social skills development activities and then find out what works best for a certain child. It would be an important objective for NAO to learn child preferences from session to session and adapt its behaviors accordingly.

Impact of COVID-19 on HRI: If we consider the significant decrease in an experiment-based HRI, it becomes clear that some of us may not embrace an online research environment. There might be a serious disparity between subject areas, institutional support, and early-career and expert researchers. Besides, there is a geographical factor that might influence research activity as some countries (e.g. Israel, New Zealand) cope better with COVID-19, while others (e.g. USA, Italy) have been hardest hit by it. Thus, a collaboration between researchers within and beyond the field can be a silver lining of current research-related challenges.

## 8 Conclusion and Limitations

In HRI, we often work and develop closer ties with a particular robot, and may overlook how other robots contribute to the field. In this review, we presented a comprehensive overview on the use of NAO, which is a remarkable social robot in many instances. So far, NAO has been exposed to challenging yet rewarding journey. Its social roles have expanded thanks to its likeable appearance and multi-modal capabilities followed by its fitness to deliver socially important tasks. Still, there are gaps to be filled in view of sustainable and user-focused human-NAO interaction. We hope that our review can contribute to the field of HRI that needs more reflection and general evidence on the use of the social robots, such as NAO in a wide variety of contexts. The main limitation to this study is that our search was limited to keywords in abstract and titles. It means that we could not cover other studies that might enrich the current review. Nevertheless, we believe that our research may engender important insights into the use of NAO across different domains and shape a broader understanding of human-robot interaction over the last decade. An implication of the findings shows a greater need for increasing the value and practical application of NAO in user-centered studies. Future studies should consider the importance of real-world and unrestricted experiments with NAO and involve other humans that might facilitate human-robot interaction.

## Data Availability

The dataset generated for this study can be found in the Zenodo repository https://zenodo.org/record/5576799.

## Footnotes

1
http://www.aliz-e.org/

2
http://dream2020.eu/

3
http://chili.epfl.ch/cowriter

4
http://www.squirrel-project.eu/

5
http://www.l2tor.eu/

6
https://www.asknao-tablet.com/en/home/

7
https://www.robotlab.com/store/zora-robot-solution-for-healthcare

8
https://www.scopus.com

9
https://humanrobotinteraction.org/2021/full-papers/

10
https://zenodo.org/record/2567595#.YPVq0y8RoUs

11
https://www.generationrobots.com/en/403100-programmable-humanoid-robot-nao-v6.html

12
https://www.naoteamhumboldt.de/en/team/
